# Generation of hypoxia-sensing chimeric antigen receptor T cells

**DOI:** 10.1016/j.xpro.2021.100723

**Published:** 2021-08-06

**Authors:** Paris Kosti, Karen I. Larios-Martinez, John Maher, James N. Arnold

**Affiliations:** 1 School of Cancer and Pharmaceutical Sciences, King’s College London, Faculty of Life Sciences and Medicine, Guy’s Campus, London SE1 1UL, UK; 2 Department of Immunology, Eastbourne Hospital, Kings Drive, Eastbourne, East Sussex BN21 2UD, UK; 3 Department of Clinical Immunology and Allergy, King’s College Hospital NHS Foundation Trust, Denmark Hill, London SE5 9RS, UK

**Keywords:** Cell culture, Cell isolation, Flow cytometry/mass cytometry, Cell-based assays, Cell separation/fractionation, Cancer, Immunology, Molecular biology, Biotechnology and bioengineering

## Abstract

Exploiting hypoxia in solid malignancies to restrict expression of chimeric antigen receptors (CARs) on engineered T cells to the tumor microenvironment overcomes the risk of on-target off-tumor toxicity and minimizes tonic signaling, which promotes CAR T cell exhaustion. This protocol summarizes the synthetic biology underlying the development of a stringent oxygen-sensitive CAR for *in vitro* and *in vivo* preclinical characterization.

For complete details on the use and execution of this protocol, please refer to Kosti et al. (2021).

## Highlights

• Hypoxia can be exploited as a selective signal for tumor-specific CAR expression
• A protocol to enable stringent hypoxia-dependent CAR expression
• A dual oxygen-sensing expression system that is both dynamic and tunable
• An approach to provide a safety switch for non-tumor selective CAR targets

## Graphical abstract

**Figure:**
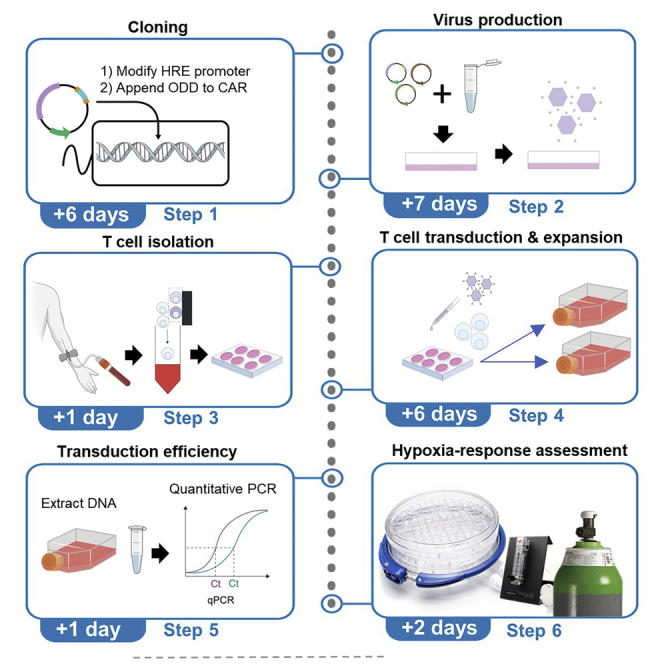

## Before you begin

***Note:*** This protocol describes the steps taken to express a generic chimeric antigen receptor (CAR) under a ‘dual oxygen-sensing’ expression system termed ‘HypoxiCAR’ using a Moloney murine leukemia (MoMLV) retroviral vector, SFG, as a means to achieve selective CAR and/or transgene expression in the tumor microenvironment when transduced into T cells. Although the approach can be expected to be applicable to other transgene carrier vectors (for example lentiviral vectors) and immune cell types, these have not been directly explored or optimized.

***Note:*** Experimental results of the ‘single’ and ‘dual’ oxygen-sensing system used with a CAR, which are presented in this protocol, refer to a pan-ErbB-specific CAR designated T1E28z. T1E28z is a CD28-containing second generation CAR with specificity for eight distinct ErbB dimers found in many cancer types (Davies et al., 2012). Target recognition is achieved using the T1E peptide, which is a pan-ErbB ligand derived from transforming growth factor-α and epidermal growth factor (Stortelers et al., 2002). Selective expansion of retrovirus-engineered T1E28z^+^ T cells is achieved by co-expression of the CAR with the 4αβ receptor in a bicistronic single cassette separated from the CAR by a T2A sequence. 4αβ is a chimeric cytokine receptor in which the ectodomain of the IL-4 receptor α subunit has been fused to the transmembrane and endodomain of the shared IL-2/15 receptor β chain (Wilkie et al., 2010). Addition of IL-4 to T cells that co-express T1E28z and 4αβ (combination dubbed ‘T4’) permits the selective enrichment of only the gene-modified cells (Wilkie et al., 2010).

***Note:*** Oxygen-sensing, as described here, relies on Hypoxia inducible factor (HIF)1α stabilization in the cell of interest when placed under limiting oxygen concentrations (typically <5% O_2_). Various cells/cell lines respond differently to changes in oxygen tension, and changes in HIF1α levels upon hypoxia exposure may consequently vary. Therefore, if the cell of interest is not a T cell, it is suggested to measure HIF1α levels under normoxic and hypoxic conditions in the cell/cell line to ensure that HIF1α is both completely degraded under conditions of normoxia and also efficiently stabilized under conditions of hypoxia. This can be assessed using a western blot approach.

***Note:*** As examples for the protocol below we present data generated using the T-47D estrogen receptor positive breast cancer cell line and/or primary human T cells (isolated and cultured as described below). HIF1α levels in T-47D and human T cells are very low/non-detectable in normoxia and are significantly stabilized upon 18 h exposure to 0.1% O_2_ (data not shown).

***Note:*** For assessing the transduction efficiency of the CAR T cells, one potential option is to use quantitative-PCR. The primers designed amplify a sequence within the *Thosea Asigna* (T2A) (Szymczak et al., 2004) ribosomal skip peptide cDNA (that is incorporated into the T4 construct to allow post-translational cleavage of the CAR and 4αβ elements) and a host cell genomic reference which was within the *TBP* gene, which encodes human TATA box binding protein. The user should evaluate if the T2A primer set is suitable for their construct (if a T2A sequence has or could be used) or alternatively design primers for their transgene of interest.

## Key resources table

**Table:** 

REAGENT or RESOURCE	SOURCE	IDENTIFIER
**Antibodies**		

Anti-human CD3ε Brilliant Violet 421 (clone SK7, 1:100 dilution)	BioLegend	Cat# 344834; RRID:AB_2565675
Anti-human CD8α Alexa Fluor 488 (clone RPA-T8, 1:100 dilution)	BioLegend	Cat# 301021; RRID:AB_2561281
Anti-Human CD4 PE (clone RPA-T4, 1:100 dilution)	eBioscience	Cat# 12-0049-42, RRID:AB_1582249
Anti-Human/Primate EGF Biotinylated (reconstituted 1 mg/mL PBS, used 1:100 dilution)	R&D Systems	Cat# BAF236; RRID:AB_356307
Streptavidin APC (1:100 dilution)	BioLegend	Cat# 405207
7-aminoactinomycin D (reconstituted 1 mg/mL PBS, used 1:1000 dilution)	Cayman Chemical Company	Cat# 11397

**Bacterial and virus strains**		

Stbl3 *E. coli*	Thermo Fisher Scientific	Cat# C737303

**Biological samples**		

Human blood/T-cells	Healthy volunteers	West of Scotland Research Ethics Committee 3 (REC reference 18/WS/0047)

**Chemicals, peptides, and recombinant proteins**		

Ficoll-Paque PLUS	GE Healthcare	Cat# GE17-1440-02
RPMI 1640	Thermo Fisher Scientific	Cat# 11875093
IMDM	Thermo Fisher Scientific	Cat# 21980032
DMEM	BioScience	Cat# BE12-604Q
Fetal Bovine Serum	Sigma-Aldrich	Cat# F7524-500ML
Human Serum	Sigma-Aldrich	Cat# H4522-100ML
LB Broth, Miller	Fisher Bio Reagents	Cat# BP9723-500
Nuclease-free water	Invitrogen	Cat# AM9938
T4 DNA ligase	Thermo Fisher Scientific	Cat# EL0011
RetroNectin recombinant human fibronectin fragment	Takara	Cat# T100B
Dynabeads human T-Activator CD3/CD28	Thermo Fisher Scientific	Cat# 11131D
Restriction endonucleases	New England Biolabs	Various, as per this paper
FuGENE HD Transfection Reagent	Promega	Cat# E2311
Recombinant Human IL-4	PeproTech	Cat# 200-04
Proleukin (aldesleukin), human recombinant IL-2	Clinigen Group	N/A
KiCqStart SYBR green qPCR ReadyMix, with ROX	Sigma-Aldrich	Cat# KCQS02

**Critical commercial assays**		

TaqMan Gene Expression Assay	Thermo Fisher Scientific	Cat# 4331182
QIAGEN Plasmid Mini, Midi and Maxi Kits	QIAGEN	Cat# 12125, 12145, 12163
QIAGEN DNeasy Blood & Tissue Kit	QIAGEN	Cat# 69506
Pan T Cell Isolation Kit, human	Miltenyi Biotec	Cat# 130-096-535

**Experimental models: Cell lines**		

T-47D	ATCC	Cat# HTB-133
HEK293T	ATCC	Cat# CRL-3216; RRID:CVCL_0063

**Oligonucleotides**		

Synthetic DNA gene fragments	Integrated DNA Technologies	Various, sequences shown Figures S1 and S2
TTTGGTGTTTGCTTCAGTCAG	Integrated DNA Technologies	Fwd TBP primer for quantitative PCR
ATACCTAGAAAACAGGAGTTGCTCA	Integrated DNA Technologies	Rev TBP primer for quantitative PCR
CGGAGAAAGCGCAGC	Integrated DNA Technologies	Fwd T2A primer for quantitative PCR
GGGTCCGGGGTTCTCTT	Integrated DNA Technologies	Rev T2A primer for quantitative PCR

**Recombinant DNA**		

SFG CBG99Luc-P2A-EGFP	This study	P1
SFG HRE9 CBG99luc-ODD401-603-P2A-GFP	This study	P20; HypoxiLuc reporter
SFG 4αβ-2A-T1E-CD28-CD3z-ODD	This study	P22
SFG HRE9 4αβ-2A-T1E-CD28-CD3z	This study	P26
SFG HRE9 4αβ-2A-T1E-CD28-CD3z-ODD	This study	P23; HypoxiCAR

**Software and algorithms**		

FlowJo v.10 Software	Tree Star	https://www.flowjo.com/
Prism 9	GraphPad	https://www.graphpad.com/scientific-software/prism/
SnapGene	GSL Biotech	https://www.snapgene.com/

**Other**		

Hypoxia Incubator Chamber	STEMCELL Technologies	Cat# 27310
Peq-Pam 3 Plasmid	n/a	n/a
RDF Plasmid (encodes RD114 envelope)	n/a	n/a
MicroAmp™ Optical 96-Well Reaction Plate	Thermo Fisher Scientific	Cat# n8010560
Adhesive PCR Plate Seals	Thermo Fisher Scientific	Cat# AB0558
Gas cylinders	BOC	Custom - using variable O_2_ as indicated_,_ 5% CO_2_ and N as balance

## Materials and equipment

**Table:** 

R5 medium (store at 4°C)	Final concentration	Amount
RPMI 1640	-	475 mL
Human serum	5%	25 mL
**Total**	**n/a**	**500 mL**

I10 medium (store at 4°C)	Final concentration	Amount
IMDM	-	450 mL
Fetal bovine serum	10%	50 mL
**Total**	**n/a**	**500 mL**

**Table:** 

D10 medium (store at 4°C)	Final concentration	Amount
DMEM	-	450 mL
Fetal bovine serum	10%	50 mL
**Total**	**n/a**	**500 mL**

Quantitative-PCR MasterMix (store −20°C)	Final concentration	Amount (for 20 μL reaction)
KiCqStart SYBR green qPCR ReadyMix (2×)	1×	10 μL
Forward primer	200 nM	Variable
Reverse primer	200 nM	Variable
Nuclease-free water	Variable	add to 20 μL
Template	5 ng/mL	Variable
**Total**	**n/a**	**20 μL**

***Alternatives:*** In addition to SYBR Green, other DNA-binding dyes are available commercially which can be used such as EvaGreen dyes. Select your reagents according to your equipment availability.

## Step-by-step method details

### Modifying a retroviral vector to contain a hypoxia-responsive promoter

**Timing: +3 days (depending on sequencing service)**

This protocol describes how to modify the SFG retroviral vector to contain a hypoxia-responsive promoter. To achieve this, the enhancer region of the 3′ long terminal repeat (LTR) in the SFG vector is modified to contain a poly-hypoxia-responsive elements (HRE) sequence.

**Figure 1:**
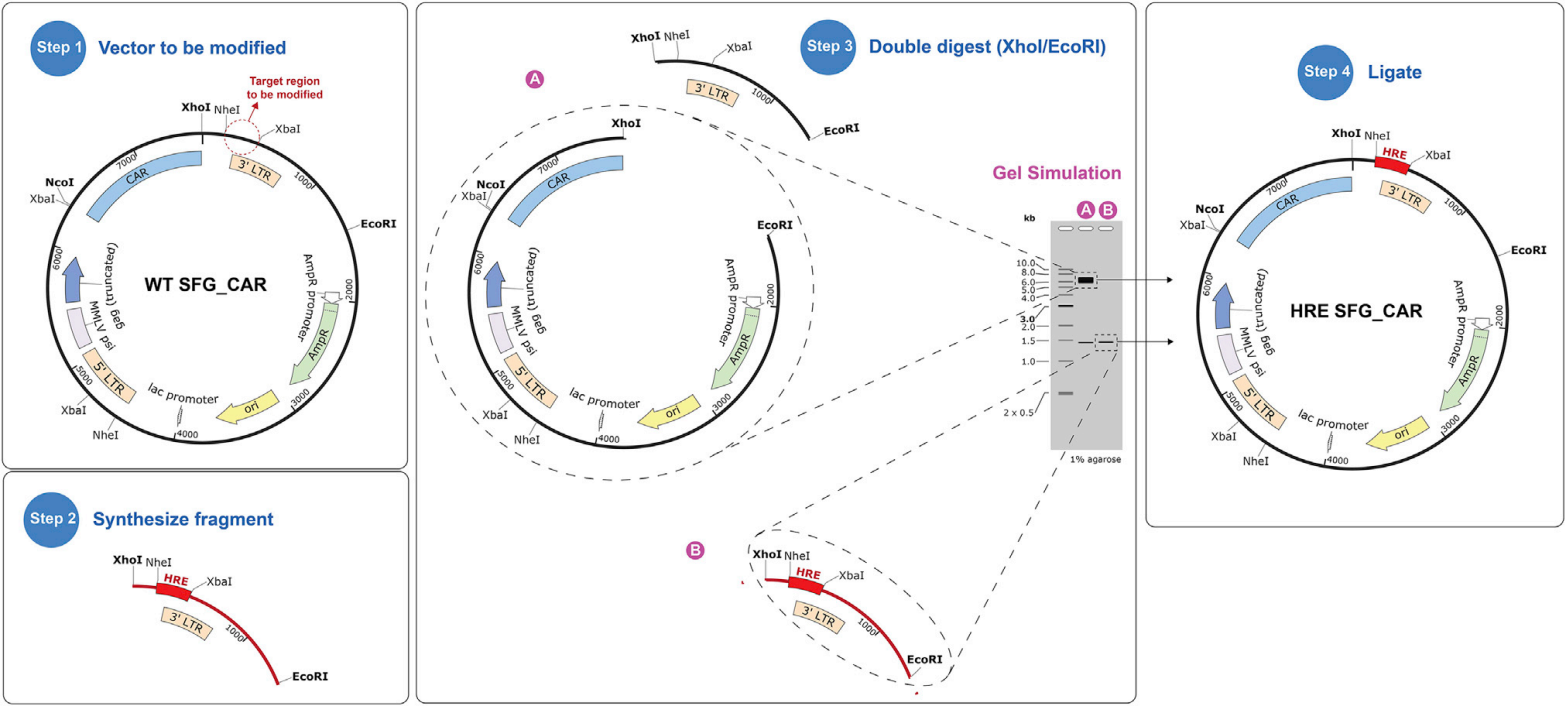
Overview of the cloning steps to modify the promoter of a CAR (1) Identification of the locus on the vector to be modified. (2) Synthesize fragment containing the HRE repeats. (3) Use restriction endonucleases to double digest the SFG vector and the HRE fragment and recover DNA from gel using a DNA ladder to guide band identification. (4) Ligate SFG backbone (A) and HRE fragment (B).

**Figure 2:**
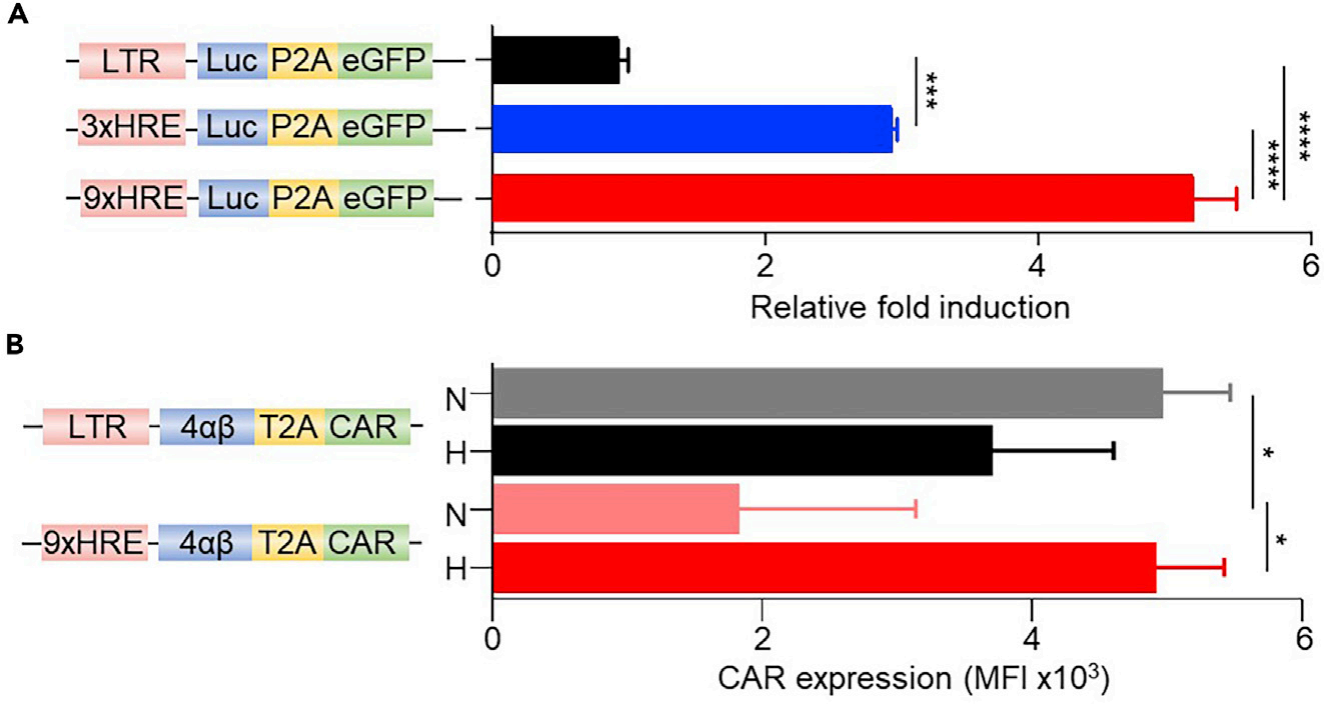
A tandem-HRE promoter provides tuneable but leaky hypoxia induction of a transgene (A) Tandem HRE repeats (3× or 9×) were modified in the SFG vector encoding a reporter transgene of Click beetle luciferase (Luc) and enhanced green fluorescent protein (eGFP; schematic on the left) and transduced in T-47D cells. After exposure for 18 h under either hypoxic (0.1% O_2_) or normoxic (20% O_2_) conditions, cells were assessed for their respective luciferase activity, presented as fold induction between the two conditions (n = 4-8). (B) Human T cells were stably transduced with the indicated CAR constructs (schematic on the left) and exposed for 18 h under either hypoxic (0.1% O_2_) (H) or normoxic (20% O_2_) (N) conditions prior to assessment of their surface CAR expression/cell presented as median fluorescence intensity of staining/cell (MFI) using flow cytometry analyses (n = 3). RLU value recorded was divided by the relative transduction efficiency for each construct (to normalize to the respective transduction efficiency). Bars represent mean and error bars represent S.D. ∗ p<0.05, ∗∗∗ p<0.001, ∗∗∗∗ p<0.0001.

***Note:*** Modification should be targeted to the 3′ LTR as this will be replicated to the 5′ LTR position upon integration of the provirus genetic material into the recipient host cell DNA (Levin et al., 2010). HREs can be derived from genes that are upregulated in response to hypoxia (Liu et al., 1995). Here we use HREs from human erythropoietin gene (EPO; Gene ID: 2056). We utilized a nine tandem HRE construct which is incorporated into the 3′ LTR using the XhoI/EcoRI restriction endonuclease cleavage sites (Figure 1). Although increasing the number of tandem HREs are likely to further increase the strength of the promoter in hypoxia, a 9xHRE cassette was selected as it had an approximate base pair length to the enhancer region removed. However, it should be noted that the strength of the promoter is tunable, and the utilization of fewer HRE tandem repeats results in a weaker fold induction of the transgene (Figure 2A).

1. Identify the locus in the SFG 3′ LTR enhancer region to be replaced for a hypoxia-responsive promoter.
2. Synthesize the HRE fragment with the desired number of tandem HRE repeats as a synthetic DNA fragment (the 9xHRE fragment sequence is shown in Figure S1) with XhoI and EcoRI restriction sites flanked by at least 6 b.p. overhangs to allow efficient cleavage. These overhang b.p. are a random sequence, however, the extra b.p. should be chosen so that palindromes and primer dimers are not formed.

***Note:*** Synthetic DNA fragments can be ordered from Integrated DNA Technologies (IDT), GenScript, or others.

***Alternatives:*** Besides synthetic DNA fragments other cloning methods can be used such as overlapping PCR and HiFI.

3. Double restriction endonuclease digest your construct containing the SFG backbone and your synthetic DNA fragment containing the HRE repeats.
  a. Set the following reaction for the vector and backbone in separate tubes:

**Table:** 

Component	Quantity
DNA to be digested	1 μg
10× CutSmart Buffer	5 μL (1×)
XhoI	1 μL (20 units)
EcoRI-HF	1 μL (20 units)
Nuclease-free water	Up to 50 μL
  b. Incubate at 37°C for 1 h.
    ***Note:*** Enzyme volume should not exceed 10% of the total reaction volume to prevent non-specific restriction activity. For enzyme-specific digestion protocols refer to: http://nebcloner.neb.com/#!/redigest

4. Add Gel Loading Dye, Purple (6×) to the digestion products; 10 μL for a standard 50 μL reaction.
5. Run the digested sample containing loading dye on a 1% agarose gel containing 0.5 μg/mL ethidium bromide, for the duration needed to observe a clear separation of the 2 bands in the digested SFG vector backbone (Figure 1).
6. Cut the individual bands of interest from your gel (Figure 1) using a scalpel on a UV light box.

**CRITICAL:** Minimize the exposure of UV light on your gel while imaging on a UV light box to avoid DNA damage.

7. Recover the DNA from the excised gel bands using QIAquick Gel Extraction Kit, according to manufacturers’ handbook: https://www.qiagen.com/ch/products/discovery-and-translational-research/dna-rna-purification/dna-purification/dna-clean-up/qiaquick-gel-extraction-kit/

***Alternatives:*** Other kits are available, for example, Monarch® DNA Gel Extraction Kit.

***Note:*** At this stage, the SFG backbone fragment and the HRE fragment bear flanking compatible sticky ends that can be annealed.

8. Ligate the digested SFG backbone (7,516 b.p.) and the digested HRE synthetic DNA fragment (1,480 b.p.) together using T4 DNA Ligase, at a molar ratio of 3:1 insert to vector, according to manufacturers’ protocol: https://international.neb.com/protocols/0001/01/01/dna-ligation-with-t4-dna-ligase-m0202
9. Transform One Shot™ Stbl3™ chemically competent *E. coli* with the ligation mix and spread transformed bacteria on a Luria broth (LB) agar plate containing 100 µg/mL ampicillin for selection as per manufacturers’ protocol: https://www.thermofisher.com/order/catalog/product/C737303#/C737303

***Note:*** It is recommended to perform a parallel control ligation in which nuclease-free water, in place of the insert, is added to SFG backbone. This control ligation mixture can then be transformed as described above and can serve as a negative control in the transformation reaction, thereby giving the background signal. Although the gel-purified SFG backbone is not expected to re-ligate as it is flanked by incompatible sticky ends (XhoI and EcoRI), this negative control reaction ensures that the SFG backbone was completely digested and specifically recovered from the gel. In principle, this control should be free of colonies, but in reality, it may have some amount of background (for example from contamination with uncut plasmid). Ideally, the SFG backbone and insert ligation should, at least, have substantially more colonies than the vector and water control ligation.

10. When bacterial colonies are visible on the LB agar plate, select a few colonies to grow up in 3 mL cultures of LB media containing 100 μg/mL ampicillin 16 h at 37°C, with agitation.
11. Purify plasmid using a Mini Prep kit (Qiagen) and analyze the purified vectors by sequencing to confirm that the correct plasmid has been generated, from now on named SFG-HRE.

***Note:*** The resulting SFG-HRE construct, is leaky for the expression of transgenes (including a CAR) in normoxia (Figure 2), but is used as the base vector into which an oxygen-dependent degradation domain (ODD)-appended transgene is cloned to create the stringent dual hypoxia-dependent CAR expression system.

### Modifying a CAR to contain an oxygen-dependent degradation domain

**Timing: +3 days (depending on sequencing service)**

**Figure 3:**
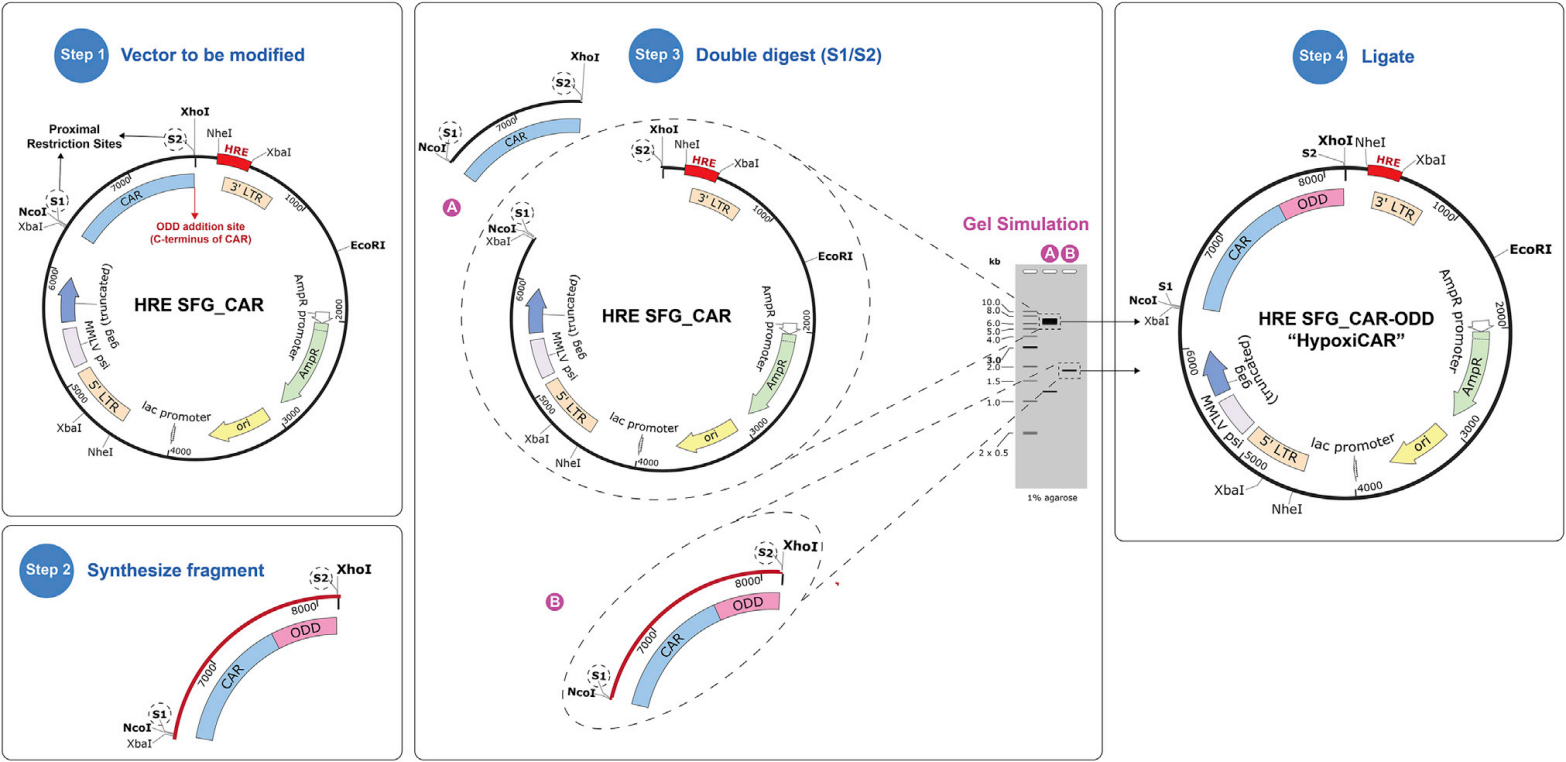
Overview of the cloning steps to append an ODD to a CAR (1) Identify locus on the vector (SFG-HRE) to be modified along with proximal unique restriction sites (in this case NcoI/XhoI). (2) Synthesize fragment containing the ODD flanked by sequence identical to the CAR sequence including the proximal restriction sites identified (NcoI/XhoI) and at least 6 additional random b.p. overhangs (to allow efficient restriction endonuclease cleavage) to either side. (3) Double digest the SFG-HRE CAR and the ODD-containing synthetic fragment and recover DNA from gel (fragments to be recovered from gel are indicated). (4) Ligate DNA fragments (A) and (B).

This section describes how to append an ODD to a CAR. This is achieved by directly fusing a human ODD (a.a. residues 401–603) from HIF-1α to the C-terminus of the CAR CD3ζ cytoplasmic domain (Figure 3).

**Figure 4:**
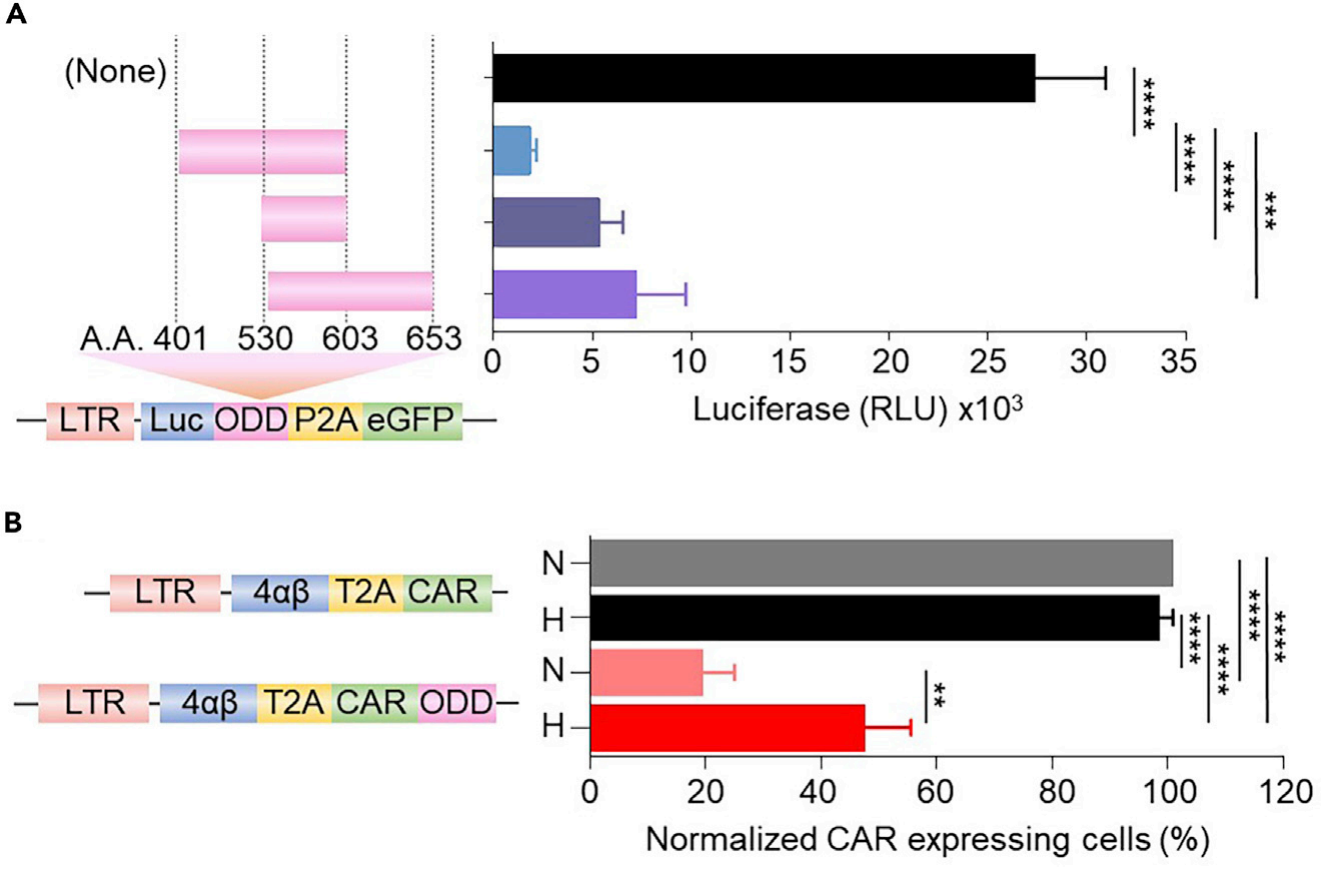
An ODD partially destabilizes a CAR in normoxic conditions (A) Three human HIF1α-derived ODD residues (a.a. residues 401–603, 530–603, and 530–653, schematic on left of Figure) were appended to the luciferase gene in the reporter SFG vector and transduced in T-47D cells. After exposure for 18 h under normoxia, cells were assessed for their respective luciferase activity, presented as RLU. (n = 3-7). (B) Human T cells were stably transduced with a SFG vector containing T4-CAR or T4-CAR-ODD (a.a. residues 401–603) (schematic on the left) and exposed for 18 h under either hypoxic (H) or normoxic (N) conditions prior to assessment of their surface CAR expression presented as percentage of normalized CAR expressing cells relative to the non-ODD appended CAR under conditions of normoxia using flow cytometry analyses (n=3). Values were normalized to the relative transduction efficiency of each construct as assessed by qPCR to enable accurate comparisons across constructs. Bars represent mean and error bars represent S.D. ∗∗: p<0.01, ∗∗∗: p<0.001, ∗∗∗∗: p<0.0001.

***Note:*** If space in the vector is limited, a smaller fragment of the ODD could be used (Figure 4A). In our initial optimization, we directly appended three potential human HIF-1α-derived ODD residues: a.a. residues 401–603, 530–603, and 530–653. All three ODDs resulted in a significant destabilization of the protein of interest under conditions of normoxia (Figure 4A) and conferred stabilization of the fusion partner in hypoxia (data not shown).

12. Identify the locus on the vector (SFG-HRE) to be modified along with two proximal unique restriction sites (one upstream and one downstream as the multiple cloning site can vary). In our SFG-HRE CAR vector we chose to append the ODD on the C-terminus of the CAR directly after the CD3ζ intracellular domain, and selected the NcoI and XhoI in the multiple cloning site of the SFG vector as proximal unique restriction sites.
13. Synthesize the ODD fragment to be appended to the C-terminus of the CAR as a synthetic DNA fragment (can modify sequence shown in Figure S2).

***Alternatives:*** Different lengths of ODDs can be used as stated before (Figure 4A). Although not tested in this system, ODDs can be also placed at the N-terminus of a protein of interest (Smirnova et al., 2010) and/or linkers can be interposed between for flexibility and/or additional spacing from the fusion partner. Linkers may be used in case the ODD interferes with the function of its fusion partner.

14. Double restriction endonuclease digest your HRE-modified SFG vector and your synthetic DNA fragment containing the CAR-ODD using NcoI and XhoI enzymes.
  a. Set the following reaction for the fragment and vector in separate tubes:

**Table:** 

Component	Quantity
DNA to be digested	1 μg
10× CutSmart Buffer	5 μL (1×)
NcoI-HF	1 μL (20 units)
XhoI	1 μL (20 units)
Nuclease-free water	Up to 50 μL

    ***Note:*** Volumes and masses may be needed to optimize, depending on the construct being used.
  b. Incubate at 37°C for 1 h.

15. Repeat steps 4 to 11 on the SFG-HRE vector and CAR-ODD insert. The bands of interest are the digested SFG-HRE backbone (6,391 b.p.) and the digested ODD synthetic DNA fragment to be used as the insert (1,824 b.p.) (Figure 3). The resulting construct containing the dual hypoxia-sensing system (HRE promoter and ODD) will be referred to herein as ‘HypoxiCAR’.

***Note:*** The resulting HypoxiCAR construct can then be used for retrovirus production and transduction of T cells as described in the sections below.

### Transfection - transient virus production

**Timing: 7 days**

**Figure 5:**
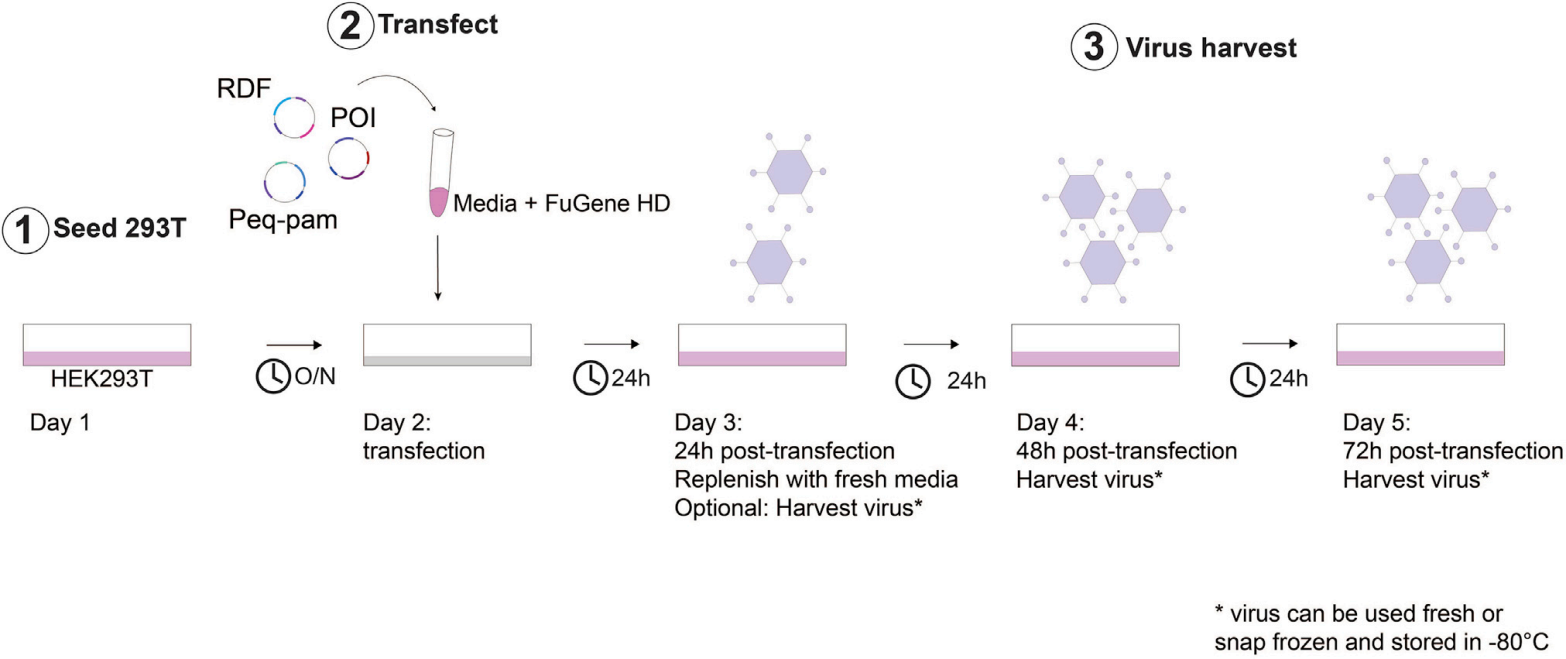
Workflow of virus production (1) HEK 293T cells are seeded 1 day prior to transfection. (2) Transfection process is performed. (3) Media is replaced (optionally virus-containing supernatant can be harvested and used or stored as indicated). (4) 48 h viral supernatant is harvested. (5) 72 h viral supernatant is harvested. POI: plasmid of interest (i.e., HypoxiCAR construct); O/N: overnight 16 h culture.

The step of transfection allows the introduction of plasmid DNA into eukaryotic cells. This protocol uses FuGENE Transfection Reagent, a non-liposomal formulation, to introduce DNA into HEK293T cells. This method results in transient expression of the vector for retrovirus production (Figure 5).

***Note:*** There are also other protocols to efficiently produce retroviral particles carrying the transgene of interest. Should such protocols already be established in users’ laboratory, if preferred, these can be used to generate transgene-carrier retrovirus.

16. At least four days before the transduction:
  a. Thaw a vial of HEK293T cells in the water bath at 37^°^C.
    ***Note:*** Transfection can also be performed with HEK293T cells kept in culture. However low passages of this cell line often produce higher titers of transient virus, leading to higher transduction efficiencies.
  b. Seed the content of the HEK293T vial (approximately 1 × 10^7^ cells in 1 mL) in a T75 flask in 25 mL 37^°^C pre-warmed I10 medium.
  c. Place flask into incubator at 37°C and 5% CO_2_.
  d. Check for confluency on ensuing days.

17. One day before the transfection:
  a. Prepare a suspension of HEK293T cells at 2.3 × 10^5^ cells/ mL in I10.
  b. Add 11 mL of the HEK293T suspension to a 100 mm plate, for a total of 2.5 × 10^6^ cells per plate.
    ***Note:*** One plate is required per construct. One 100 mm plate of HEK293T cells will produce around 20 mL of viral supernatant (2 harvests), enough to transduce 4 × 10^6^ human T cells. This step can be downscaled or upscaled as required.
  c. Gently rock the plate to ensure aneven distribution of cells across the plate.
  d. Place plate into incubator.

18. The day of the transfection, per 100 mm plate:
  a. In a 1.5 mL Eppendorf tube add 1.133 mL of IMDM media and 68 μL of FuGENE reagent.
  b. In another 1.5 mL Eppendorf tube mix the 5.6 μg RDF (RD114-encoding) envelope plasmid, 8.5 μg Peq-Pam-env (encoding gag and pol) and 8.5 μg of the vector plasmid of interest (ideally with a total volume of <100 µL).
  c. Mix the contents of the tubes in steps 18a and 18b and incubate at 20°C–22°C for 15 min.
  d. Add the mixture dropwise to the HEK293T cell plate and gently rock the plate to evenly distribute the mixture.
  e. Place the plate back into the incubator.
19. Twenty four hours after transfection:
  a. Remove and discard media from the plate and replace with 11 mL of prewarmed fresh I10 media.
  b. Place plate back into the incubator.

***Note:*** Replace the media gently, trying not to detach the transfected 293T cell monolayer.

20. Forty eight hours post transfection:
  a. Harvest the media (containing the viral particles produced) into a 50 mL Falcon tube and store at 4°C. Replace with 11 mL of warm fresh I10 media.
  b. Return the plate to the incubator.
21. Seventy two hours post transfection:
  a. Harvest the virus-containing media into a 50 mL Falcon tube and store at 4°C.
  b. Filter the harvested 48 and 72h media using a 0.45 μm sterile filter. The filtered viral supernatants can be used for T cell transduction or snap-frozen in liquid nitrogen and stored at −80°C until required.

***Note:*** Retrovirus titer gradually drops upon storage at 4°C and it is thus recommended not to store retrovirus at 4°C for extended time periods (more than 1 day) prior to use or freezing.

***Note:*** Virus-containing media supernatant harvested at 48 h or 72 h post-transfection can be also filtered separately on respective harvest day as indicated in 20 and 21 and used fresh or snap frozen for storage (if pooling of 48 h and 72 h viral supernatant is not desired).

***Note:*** Filtering viral supernatant using 0.45 μm sterile filters can cause significant viral particle loss. In place of this, the user can opt to centrifuge the virus-containing media at 500 × *g* for 5 mins to pellet any contaminating cells or debris and then remove the viral supernatant (being careful not to disturb any pelleted material) to a fresh tube before snap freezing or using fresh.

### Peripheral blood mononuclear cell isolation

**Timing: 3 h**

**Figure 6:**
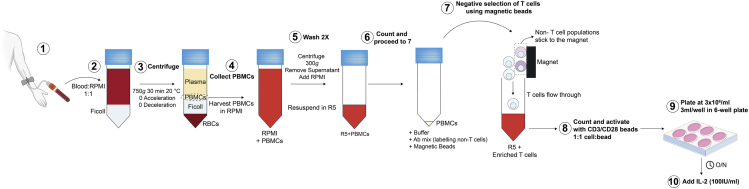
Purification of T cells from peripheral blood mononuclear cells (1–6) Peripheral Blood Mononuclear Cell (PBMC) Isolation. (1) Peripheral blood withdrawal. (2–3) Ficoll density gradient centrifugation to separate blood components. (4) Harvest PBMC layer using sterile Pasteur pipette and transfer in RPMI. (5) PBMCs are washed twice in RPMI and resuspended in R5 media. (6) Cell counting. (7) Negative selection of T cells using magnetic beads. (8–10) T cells are counted and activated as shown.

This section describes how to isolate peripheral blood mononuclear cell (PBMCs) from human blood by Ficoll gradient centrifugation (Figure 6).

22. Withdraw peripheral blood (typically 50 mL) from a healthy donor with informed consent.
23. Transfer the blood from the collection tubes to 50 mL Falcon tubes.
24. Dilute the blood 1:1 with RPMI media (in 50 mL Falcon tubes or another sterile vessel to accommodate the total volume).
25. In another 50 mL Falcon tube, add 15 mL of Ficoll-Paque Plus.
26. Carefully transfer 30 mL of the diluted blood to the Ficoll-Paque tubes as not to disturb the Ficoll layer.
27. Centrifuge the 50 mL tubes at 750 × *g* for 30 min at 20°C (acceleration and brake set to 0) to separate the PBMC cell fraction.
28. Transfer the PBMC layer, seen as an interface between the plasma and the Ficoll layer (Figure 6), using a sterile Pasteur pipette to a fresh 50 mL tube.

***Note:*** After centrifugation, the layer above the PBMC fraction which contains plasma can be carefully removed using a Pasteur pipette to make the PBMC layer easier to harvest.

29. In the 50 mL tube containing the PBMCs, add RPMI media to a final volume of 50 mL and centrifuge at 500 × *g* for 5 min. The acceleration and brake can be set back to 9.
30. Aspirate and discard the supernatant leaving the pelleted cells.
31. If more than one tube has been used (due to the volume size), the pellets can be resuspended in RPMI and combined and the cells are once again brought up to a final volume of 50 mL of RPMI for a second wash.
32. Centrifuge at 500 × *g* for 5 min.
33. Aspirate and discard the supernatant.
34. Resuspend the cell pellet in 10 mL of R5 media.

### T-cell isolation and activation

**Timing: 2 h**

This protocol describes how to purify T cells from the PBMC fraction using human pan T cell isolation kit (Miltenyi Biotec), a MidiMACs separator and LS columns (Miltenyi Biotec) and how to activate purified T cells using CD3/CD28 Human T-Activator Dynabeads (Gibco) (Figure 6).

35. For T cell isolation:
  a. Count the cells from the PBMC fraction and proceed according to the manufacturers’ protocol: https://www.miltenyibiotec.com/US-en/products/pan-t-cell-isolation-kit-human.html#gref
36. For T cell activation:
  b. Wash the CD3/CD28 Human T-Activator Dynabeads according to the manufacturers’ protocol: https://www.thermofisher.com/uk/en/home/references/protocols/proteins-expression-isolation-and-analysis/t-cell-activation-and-expansion/dynabeads-human-t-activator-cd3-cd28.html
  c. Resuspend the purified T cells at a concentration of 3 × 10^6^ cells/mL in R5 media.
  d. Add Dynabeads Human T-Activator CD3/CD28 at a bead-to-cell ratio of 1:1.
  e. Seed 3 mL of the T cells containing the Dynabeads (3 × 10^6^ cells/mL) per well on a TC-treated 6-well plate.
  f. Place plate into incubator.
37. After 24 h add recombinant human IL-2 at a final concentration of 100 IU/mL.

### Retroviral-mediated T cell transduction

**Timing: 3 days**

**Figure 7:**
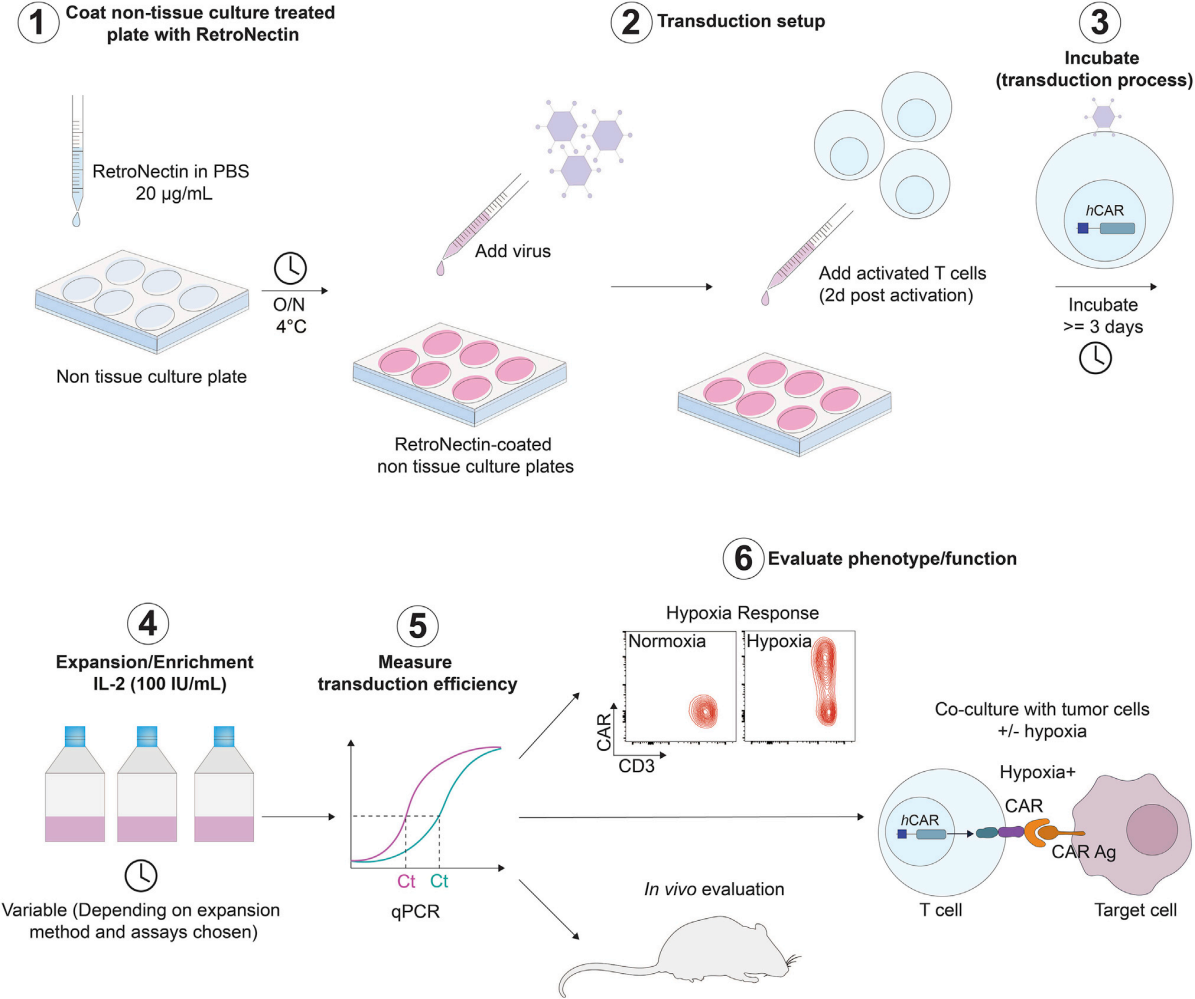
Workflow of CAR T cell production (1) RetroNectin coating. (2) Transduction setup. (3) Incubation to allow viral infection of target cells. (4) Expansion/enrichment of T cells. (5) Transduction efficiency measurement via quantitative PCR for accurate quantification of transgene abundance in the target cell population. (6) Evaluation of T cell phenotype and function (*in vitro* and *in vivo*).

This section describes the steps for retroviral-mediated transduction of human T cells. This method ensures the integration of the inserted coding DNA contained in the construct into the host T cell genome, thereby permitting stable expression of the transgene (Figure 7).

38. One day before the T cell transduction:
  a. Add 20 μg/mL RetroNectin (Takara Bio Inc.) to sterile PBS with a volume corresponding to 4 μg/cm^2^ to a non-tissue culture treated 6-well plate (2 mL of RetroNectin in PBS solution per well of a 6-well plate). Use one well per construct of interest and one for non-transduced and/or mock transduced control.
  b. Place lid on plate and wrap edges with parafilm to prevent evaporation and incubate for 16 h at 4°C.

39. On the day of the T cell transduction:
  a. Take out the RetronNectin-coated plate from the fridge and aspirate the RetroNectin/PBS solution from the well.
    ***Optional:*** Plates can be blocked using serum-containing media (2 mL per well) for 30 min at 20°C–22°C. Following blocking, media is removed.
  b. Add 2 mL of either fresh or thawed (if frozen) virus from the previous steps and incubate for 4 h at 4°C.
  c. Remove the Dynabeads from the T cells. Harvest the cells into a tube and place the tube on a magnet for 2 min to separate the beads from the solution. Transfer the supernatant containing the cells to a new tube and count the cells.
  d. Take out the virus coated plate from the fridge and remove and discard the liquid supernatant from the well.
  e. Add 3 mL of fresh or snap-frozen virus and add 1 × 10^6^ T cells in 1 mL R5 media per well.
  f. Place plate into incubator.

### CAR T-cell expansion

**Timing: +3 days (depending on the quantity of CAR T cells required)**

This section describes how to maintain and expand CAR T cells in culture (Figure 7).

40. Assess T cell growth every day after transduction.
41. On the day after the T cell transduction:
  a. Add recombinant human IL-2 into the wells at a final concentration of 100 IU/mL.
  b. Place plate into incubator.

42. Three days after T cell transduction:
  a. Gently harvest the T cells.
  b. Re-plate in a 6-well plate in fresh R5 supplemented with fresh IL-2 (100 IU/mL).
    ***Note:*** For T cells transduced with the 4αβ chimeric receptor (that is specific to T4), instead of IL-2, T cells are enriched in culture following transduction using human IL-4 (Peprotech) at 30 ng/mL final concentration (Wilkie et al., 2010). In the absence of an enrichment approach, concentrated virus can be used to improve transduction efficiencies.
  c. Place plate/flask into incubator.

***Note:*** CAR T cells ideally should be kept at a density of 1 × 10^6^ cells/mL.

43. Every two to three days:
  a. If required, add R5 media and move cells into larger plates or flasks.
  b. Add 100 IU/mL of IL-2 into the cultures.
  c. Place plate/flask into incubator.

***Note:*** It is recommended to count T cells every 2–3 days and adjust their concentration to 1 × 10^6^ cells/mL using fresh IL-2-supplemented R5 to maintain an optimal cell state during expansion.

### Assessment of HypoxiCAR T cell transduction efficiency via quantitative PCR

**Timing: 1 day**

This section describes how to assess the transduction efficiency of HypoxiCAR T cells. Since the expression of HypoxiCAR is conditional and not constitutive, standard surface staining of the CAR and subsequent flow cytometry analysis would not permit an assessment of the transduction efficiency for this approach without exposure to hypoxia (for 18 h). Therefore, quantitative PCR can be used as an assessment to determine the efficiency of transduction. To undertake this analysis, it is necessary to have identified primer pairs that specifically amplify the transgene of interest or CAR (for T4 CAR we used primer pairs specific to the T2A ribosomal skip peptide sequence) and a reference genomic housekeeping gene for which we selected *TBP*. The primer pairs we used for this analysis are listed in key resources table. These primers were used to measure stably integrated transgene abundance and hence transduction efficiency. In case T2A is absent from the SFG construct transduced (for example in non-bicistronic CAR-only SFG vectors), specific primers that bind either in the ORF (open reading frame) or in the SFG backbone can be designed and validated *in situ*. The advantage of using specific primers binding in the SFG backbone is that these can be used universally in any SFG vector transduced.

44. Extract genomic DNA from the T cell culture using a DNeasy Blood & Tissue Kit (QIAGEN) according to manufacturers’ Quick Start protocol: https://www.qiagen.com/us/products/discovery-and-translational-research/dna-rna-purification/dna-purification/genomic-dna/dneasy-blood-and-tissue-kit/

***Note:*** DNA extraction should be performed at least 4 days after retroviral transduction (to ensure stable integration of the vector). It is important for qPCR for all samples to be analyzed to use the same starting quantity DNA. Using 100 ng per sample is recommended.

45. Thaw your primers and the KiCqStart SYBR Green qPCR ReadyMix with ROX Reagent.
46. Set up your qPCR Master Mix as shown in ‘Materials and equipment’ section, with all required components except sample template (genomic DNA). Vortex gently to mix reaction.
47. Add your qPCR Master Mix to your reaction plate.
48. Add your template (genomic DNA) to your reaction plate.

***Optional:*** In additional wells, add nuclease-free water in place of genomic DNA. This reaction will serve as a negative control for the qPCR assay.

49. Seal your reaction plate using an adhesive PCR Plate seal to prevent evaporation.
50. Vortex gently to mix reaction.
51. Centrifuge plate at 500 × *g* for 1 min to collect components at the bottom of the reaction tube.
52. Place plate in an ABI 7900HT Fast Real Time PCR instrument (or equivalent compatible).
53. Set your real time-PCR cycling conditions as follows:

**Table:** 

PCR cycling conditions			
Steps	Temperature	Time	Cycles
Initial denaturation	95°C	30 s	1
Denaturation	95°C	5 s	40 cycles
Annealing	55°C	15 s	
Final extension	68°C	10 s	1

54. Collect data from the machine.
55. Data analysis uses the threshold cycle (C_t_) value of each sample. The C_t_ value represents the cycle at which the qPCR product reaches the set threshold value and hence the amount of product present.

Assumptions: This approach makes the assumption that there is equal transduction across the cell population. As opposed to flow cytometry-based quantification of transduction efficiency, the qPCR method does not provide single-cell information. Nevertheless, it provides a measurement of the abundance of the transgene (in comparison to the reference gene) in a given cell population. This value/abundance can be used to normalize data and make accurate comparisons between samples.

### Hypoxia-response assessment via flow cytometry

**Timing: 2 days**

This protocol explains how to assess the response of the HypoxiCAR to experimental hypoxia and also provides an alternative method to test transduction efficiency at the single cell level. This was performed using a hypoxic chamber followed by staining of the CAR and subsequent flow cytometry analysis.

56. Harvest T cells from the transduced and non-transduced cultures into 15 mL Falcon tubes. Assuming that the concentration of T cells is maintained at approximately 1 × 10^6^ (as recommended in this protocol), harvest at least 2.5 mLs from each T cell culture.
57. Centrifuge the tubes 500 × *g* for 5 min.
58. Remove the supernatant.
59. Resuspend T cells in 1 mL of R5 media.
60. Count cells according to your own method.
61. Adjust concentration of the cell suspension to 1 × 10^6^ cells/mL.
62. Add 1 mL of the cell suspension to a 24-well plate of your transduced constructs and non-transduced cells. Repeat this in another 24-well plate, to assess for both conditions, normoxia (20% O_2,_ 5% CO_2_, N_2_ balance) and experimental hypoxia (0.1% O_2,_ 5% CO_2_, N_2_ balance).
63. Place one of the plates inside a hypoxic chamber, on top of the polycarbonate tray.

***Note:*** Place a 20 mm Petri dish containing 10–20 mL of sterile water in the chamber under the polycarbonate tray, to prevent excessive evaporation of cultures.

**Figure 8:**
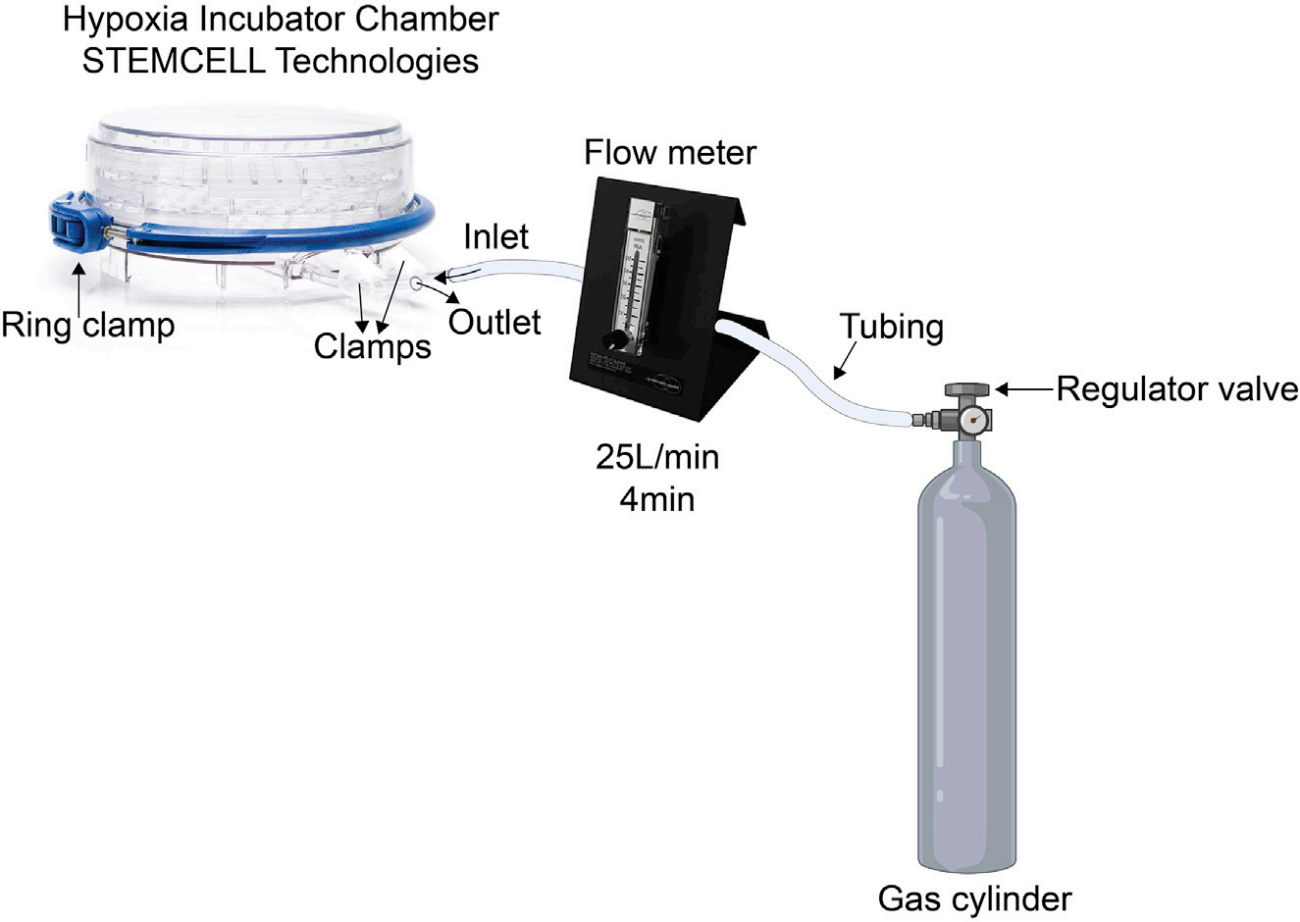
Apparatus set up for hypoxic chamber to assess hypoxia-responsive CAR expression Schematic of the components needed and set up to perform the hypoxia-response CAR T cell assessment.
66. Open both clamps for the inlet and outlet tubing of the hypoxic chamber.
67. Connect the bottom connection of the flow meter to the inlet tubing of the tank.
68. Connect the tubing coming out from the flow meter to the tube inlet of the chamber.
69. Open the gas cylinder valve.
70. Open the regulator valve control clockwise to 8–10 psi.
71. Adjust the flow meter to 25 L/min.
72. Purge the chamber with a flow rate of 25 L/min for 4 min.
73. Close the valve on the gas cylinder to prevent the gas flow.
74. Immediately close both of the clamps for the inlet and outlet tubing for the hypoxic chamber.
75. Disconnect the tubing from the chamber.
76. Turn off the valve control.
77. Place chamber into the incubator. Next to the chamber and inside the incubator place the normoxic plate.
78. After 1 h, re-purge chamber by repeating steps 66–76.
79. Place chamber into the incubator for at least 17 h.

***Note:*** For accurate comparison of hypoxia vs normoxia effect, it is recommended that the normoxic plate is placed next to the chamber when it is purged and re-purged prior to their return to the incubator. This is done to ensure that both plates are exposed to identical conditions (apart from O_2_ concentration) during the period of the assay.

64. Place the lid on the base and close the chamber properly, by closing the ring clamp.
65. Attach your system to the gas flow meter and gas cylinder (Figure 8). For reference you can also see this video: https://www.stemcell.com/technical-resources/the-hypoxia-chamber-how-to-assemble-and-purge-the-chamber.html

**Pause point:** Cultures will remain in the chamber for at least 18 hours in total, to allow sufficient amount of CAR (or the oxygen-controlled protein of interest) to be expressed.

80. Take the hypoxic chamber out of the incubator.
81. Open the outlet tubing clamp.

***Note:*** To make sure the chamber was properly sealed, a puff sound rather than a continuous flow should be audible when you open the outlet valve.

82. Open the hypoxic chamber by pulling the ring clamp to release the lid.
83. Take 200 μL of the T cell suspension from the normoxic and hypoxic conditions and add them to respective wells of a 96-well plate or Eppendorf tube for antibody staining for flow cytometry.
84. Spin the plate or tube at 500 × *g* for 5 min.
85. Remove supernatant and resuspend in 200 μL FACs buffer (PBS, 1% FBS, 0.1% sodium azide).
86. Proceed with your standard flow cytometry detection method for CAR expression using flow cytometry.

## Expected outcomes

**Figure 9:**
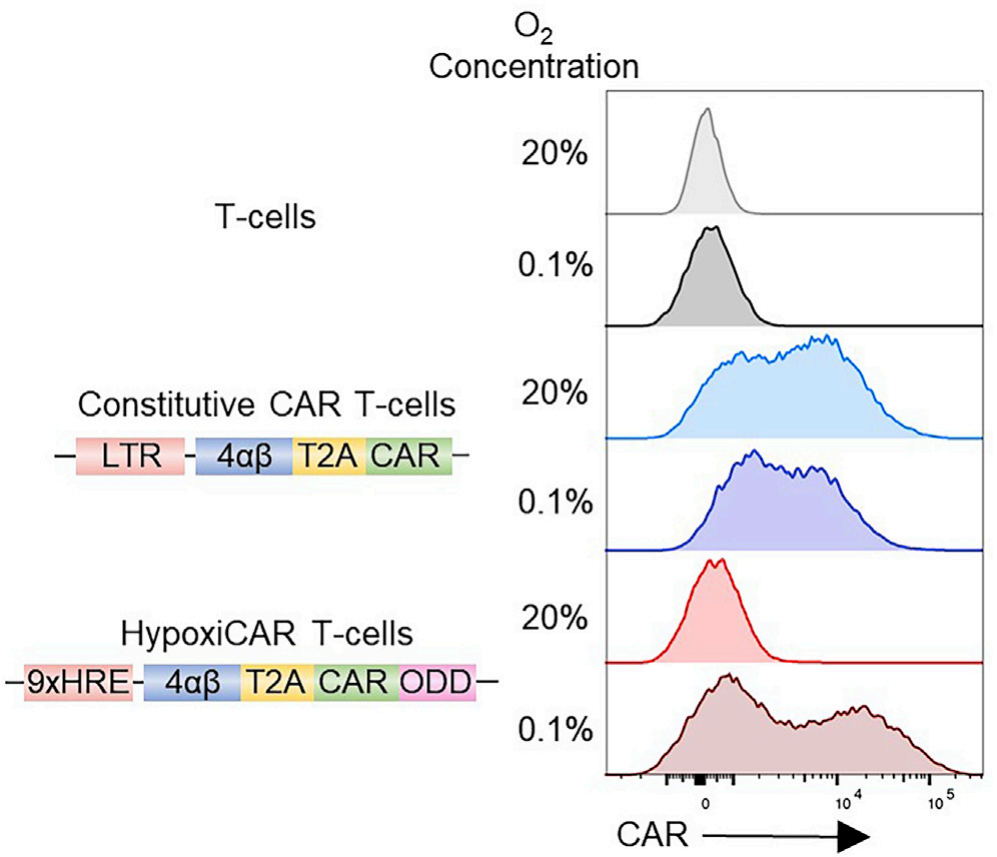
The dual oxygen-sensing expression system enables stringent oxygen-sensing CAR T cells Non-transduced human T cells or T cells stably transduced with the constitutive CAR or HypoxiCAR (HRE and ODD modifications) (schematic on the left) were exposed for 18 h in normoxic (20% O_2_) or hypoxic (0.1% O_2_) conditions prior to assessment of their surface CAR expression via flow cytometry. Representative histograms to show surface CAR expression on live (7AAD^-^) CD3^+^ T cells. Representative of multiple experiments.

It is expected that HypoxiCAR-transduced T cells will not express detectable CARs on their surface under conditions of normoxia (i.e., their CAR MFI in flow cytometry analysis should be the same or similar to non-transduced or mock-transduced T cells), while surface CAR expression in hypoxia-exposed HypoxiCAR T cells should significantly increase, potentially reaching the surface CAR expression seen in T cells transduced with the constitutive CAR construct in the same condition (i.e., hypoxia). A representative outcome histogram plot is shown in (Figure 9).

***Note:*** In a bicistronic vector system, the inherent leakiness of the HRE cassette alone (Figure 2), can be exploited to also drive expression of transgenes where it is desirable to have expression under normoxic conditions alongside a stringently regulated CAR, such as receptors which aid *ex vivo* selection (i.e 4αβ) or improve tumor targeting or penetration *in vivo*.

***Note:*** Depending on the T cell donor there may be variability in the outcomes observed, however, the HypoxiCAR induction from normoxia to hypoxia coupled with the absent or very low HypoxiCAR expression in normoxia should be expected.

## Quantification and statistical analysis

Statistical significance was determined using GraphPad Prism 9 software. Statistical analysis of construct efficacy was performed using a one-way ANOVA followed by Tukey’s post hoc tests. Homoscedasticity of residual variance and normality assumptions were met.

## Limitations

Although hypoxia represents the main stimulus for HIF1α stabilization, there are other oxygen-independent factors that can stabilize HIF1α (Majmundar et al., 2010; Palazon et al., 2014) that may thereby trigger CAR stabilization in normoxic conditions. As such, cell exposure to such factors may confound results. Although, we did not observe any hypoxia-independent stabilization of HypoxiCAR, anecdotally, we did find leakiness in the system when HypoxiCAR T cell cultures became too confluent or the media turned orange-yellow in color, indicating a lower pH.

## Troubleshooting

### Problem 1

Poor transduction efficiency. Steps 16–39.

### Potential solution 1

Too little virus may result in poor transduction efficiency and too much virus may result in toxicity in the target cell population which can also lead to poor transduction efficiencies. It is recommended to filter (0.45 μm) and concentrate virus by ultracentrifugation (24,000 × *g*, 2 h, 4°C) and subsequently titrate virus to identify the specific multiplicity of infection (MOI), or range of MOIs, that result in the optimal transduction efficiency in a specific target cell type.

### Potential solution 2

Low transfection efficiency of HEK293T cells during viral production (leading to poor virus yield). To overcome this issue, use ‘high quality’ plasmids when performing transfection. High quality plasmids can be generated using plasmid extraction kits yielding transfection-ready high-quality DNA, such as the kits developed by Thermo Fisher Scientific or Qiagen, and by carefully following manufacturers’ instructions. High quality/purity DNA should be free from phenol, sodium chloride and endotoxins. DNA contaminants can kill the cells and salt can interfere with lipid complexing, decreasing transfection efficiency. Endotoxins (released in lysis step and can be co-purified with DNA) can also reduce transfection efficiency.

Ensure that HEK293T cells are mycoplasma-free. It is also possible that the density of HEK293 T cells was too high or too low at time of transfection which generally results in poor transfection efficiency or poor viral production, respectively.

### Potential solution 3

Ensure target T cells are healthy and actively dividing at the time of transduction. In the case of human primary T cells, ensure that cells are activated and the transduction procedure is performed at an optimal time post-activation, usually within 48 h. When T cells are actively proliferating, they are often observed as clusters under a light microscope.

### Potential solution 4

The size of the vector can influence the viral yield. A solution to this is to minimize the size of the viral vector. The full HIF1α ODD domain is 203 a.a. and hence a large fragment to append on a CAR or to a protein of interest. This may negatively affect the capacity of the transgene to be packaged into functional viruses, as viruses have a limited cargo capacity. Investigators may consider using smaller ODD fragments or less HRE tandem repeats to reduce the size of the vector (Figures 2A and 4A).

### Problem 2

Leakiness in HypoxiCAR expression in normoxic *in vitro* culture. Steps 56–86.

### Potential solution

When cells become overly confluent, robust cell metabolic processes and oxygen consumption in a concentrated area in the medium may result in intermittent pericellular hypoxia (Place et al., 2017) that may induce transient HIF1α stabilization and hence HypoxiCAR expression under normoxic culture conditions. Therefore, it is recommended to maintain cell cultures at an optimal cell density (for T cells around 1 × 10^6^ cells/mL) to ensure ample oxygen supply in the media. As stated above, apart from hypoxia, there are other oxygen-independent factors that can stabilize HIF1α (Majmundar et al., 2010; Palazon et al., 2014) and hence may induce HypoxiCAR expression, however, we have not yet investigated whether and at what extent such factors affect HypoxiCAR expression.

### Problem 3

Non-responsiveness of HypoxiCAR in hypoxia chamber. Steps 56–86.

### Potential solution

To discriminate if the non-responsiveness of the HypoxiCAR T cells is CAR/donor related or due to incorrect purging or closing of the hypoxic chamber, the addition of a positive control could be considered. A retroviral packaging cell line which reliably undergoes induction of CAR or reporter expression in response to hypoxia could be used as a control. Also, it is recommended to carefully and accurately monitor oxygen levels in the hypoxia chamber, this can be done by placing an oxygen sensor spot inside the chamber and reading it through a polymer optical fiber (PreSens). Alternatively, an O_2_ Control *In Vitro* Cabinet (COY lab products) or hypoxia incubator can be used, where oxygen levels are set as desired by the user and are continuously monitored and displayed on a screen.

## Resource availability

### Lead contact

Further information and requests for resources and reagents should be directed to and will be fulfilled by the Lead Contact Dr James Arnold (james.n.arnold@kcl.ac.uk).

### Materials availability

Constructs and other reagents generated or described in this study will be made available from the Lead Contact for academic/non-commercial research purposes on request. Commercial use of the constructs generated, or derivatives, would be subject to a licensing agreement as intellectual property rights are in place.

### Data and code availability

The datasets supporting this protocol, and constructs described or used in Figures, have not been deposited in a public repository but are available from the corresponding author upon request.
