# Supplementary material for: Generation of hypoxia-sensing chimeric antigen receptor T cells
Source: STAR Protoc. 2021 Aug 6;2(3):100723. doi: 10.1016/j.xpro.2021.100723 (PMC8353489; doi:10.1016/j.xpro.2021.100723)
Supplement: Document S1. Figures S1 and S2 [file mmc1.pdf]

STAR Protocols

CellPress

# Supplementary sequences

5`-
CAGCCACTCGAGGATCCGGATTAGTCCAATTTGTTAAAGACAGGATATCAGTGGTCCAGGCTCTAGTTTTGACTCAACAATATCACCAGCTGAAGCCTATAGAGTACGAGCCATAGA
TAAAATAAAAGATTTTATTTAGTCTCCAGAAAAAGGGGGGAATGAAAGACCCCACCTGTAGGTTTGGCAAGCTAGCGGCCCTACGTGCTGTCTCACACAGCCTGTCTGACGGCCCT
ACGTGCTGTCTCACACAGCCTGTCTGACGGCCCTACGTGCTGTCTCACACAGCCTGTCTGACGGCCCTACGTGCTGTCTCACACAGCCTGTCTGACGGCCCTACGTGCTGTCTCACAC
AGCCTGTCTGACGGCCCTACGTGCTGTCTCACACAGCCTGTCTGACGGCCCTACGTGCTGTCTCACACAGCCTGTCTGACGGCCCTACGTGCTGTCTCACACAGCCTGTCTGACGGCC
CTACGTGCTGTCTCACACAGCCTGTCTGACTCTAGAGAACCATCAGATGTTTCCAGGGTGCCCCAAGGACCTGAAATGACCCTGTGCCTTATTTGAACTAACCAATCAGTTCGCTTCT
CGCTTCTGTTCGCGCGCTTCTGCTCCCCGAGCTCAATAAAAGAGCCCACAACCCCTCACTCGGGGCGCCAGTCCTCCGATTGACTGAGTCGCCCGGGTACCCGTGTATCCAATAAAC
CCTCTTGCAGTTGCATCCGACTTGTGGTCTCGCTGTTCCTTGGGAGGGTCTCCTCTGAGTGATTGACTACCCGTCAGCGGGGGTCTTTCACACATGCAGCATGTATCAAAATTAATTT
GGTTTTTTTTCTTAAGTATTTACATTAAATGGCCATAGTACTTAAAGTTACATTGGCTTCCTTGAAATAAACATGGAGTATTCAGAATGTGTCATAAATATTTCTAATTTTAAGATAGTA
TCTCCATTGGCTTTCTACTTTTTCTTTTATTTTTTTTTGTCCTCTGTCTTCCATTTGTTGTTGTTGTTGTTTGTTTGTTTGTTTGTTGGTTGGTTGGTTAATTTTTTTTTAAAGATCCTACACT
ATAGTTCAAGCTAGACTATTAGCTACTCTGTAACCCAGGGTGACCTTGAAGTCATGGGTAGCCTGCTGTTTTAGCCTTCCCACATCTAAGATTACAGGTATGAGCTATCATTTTTGGT
ATATTGATTGATTGATTGATTGATGTGTGTGTGTGTGTGATTGTGTTTGTGTGTGTGACTGTGAAAATGTGTGTATGGGTGTGTGTGAATGTGTGTATGTATGTGTGTGTGTGAGTGTG
TGTGTGTGTGTGTGCATGTGTGTGTGTGTGACTGTGTCTATGTGTATGACTGTGTGTGTGTGTGTGTGTGTGTGTGTGTGTGTGTGTGTGTGTGTGTTGTGAAAAAATATTCTATGG
TAGTGAGAGCCAACGCTCCGGCTCAGGTGTCAGGTTGGTTTTTGAGACAGAGTCTTTCACTTAGCTTGGAATTCACTGGC-3’

Annotations:

3’-LTR

Enzyme Recognition Site

NheI restriction site

XbaI restriction site

HRE Repeats (9x)

HBS (HIF-binding site)

6b.p. overhang (for efficient restriction endonuclease cleavage)

**Figure S1. Synthetic nine tandem HRE fragment sequence.** Related to Fig. 1.

**Panel A b.p. sequence**

5`-AGACTGCC**ATG**G ...
GCCCCAGCCGCTGGAGACACAATCATATCTTTAGATTTTGGCAGCAACGACACAGAAACTGATGACCAGCAACTTGAGGAAGTACCATTATATAATGATGTAATGCTCCCCTCACCC
AACGAAAAATTACAGAATATAAATTTGGCAATGTCTCCATTACCCACCGCTGAAACGCCAAAGCCACTTCGAAGTAGTGCTGACCCTGCACTCAATCAAGAAGTTGCATTAAAATTA
GAACCAAATCCAGAGTCACTGGAACTTTCTTTTACCATGCCCCAGATTCAGGATCAGACACCTAGTCCTTCCGATGGAAGCACTAGACAAAGTTCACCTGAGCCTAATAGTCCCAGT
GAATATTGTTTTTATGTGGATAGTGATATGGTCAATGAATTCAAGTTGGAATTGGTAGAAAAACTTTTTGCTGAAGACACAGAAGCAAAGAACCCATTTTCTACTCAGGACACAGAT
TTAGACTTGGAGATGTTAGCTCCCTATATCCCAATGGATGATGACTTCCAGTTACGTTCCTTCGATCAGTTGTCACCATTAGAAAGCAGTTCCGCAAGCCCTGAAAGCGCAAGTCCTC
AAAGCACAGTTACAGTATTCCAG**TAA**CAGCCACTCGAGGATCCG-3´

Annotations:

Enzyme Recognition Site

**Start codon**

... = CAR or protein of interest sequence (left to right, from N- to C-terminus)

ODD

EcoRI site (unused) and suggested b.p. to mutate to remove if it will pose an issue for downstream cloning

**Stop codon**

6b.p. overhang (for efficient restriction endonuclease cleavage)

**Panel B amino acid sequence**

APAAGDTIISLDFGSNDTETDDQQLEEVPLYNDVMLPSPNEKLQNINLAMSPLPTAETPK
PLRSSADPALNQEVALKLEPNPESLELSFTMPQIQDQTPSPSDGSTRQSSPEPNSPSEYC
FYVDSDMVNEFKLELVEKLFAEDTEAKNPFSTQDTDLDLEMLAPYIPMDDDFQLRSFDQL
SPLESSSASPESASPQSTVTVFQ

**Figure S2. ODD sequences.** Related to Fig. 3. **A**) Synthetic ODD fragment DNA sequence. The boxed codon within the purple ODD region (GAA) represented part of an EcoRI site that may cause issues should the user need to further modify the HRE cassette after incorporating the CAR-ODD into the SFG vector (as the HRE promoter insertion uses and EcoRI cleavage site). Should this potentially be needed, we recommend mutating the highlighted site to [GAG] to avoid the EcoRI site but maintain the a.a. sequence of the ODD protein when translated. **B**) Amino acid sequence of human HIF1α ODD (a.a. 401-603).
